# Commentary: on the effects of health expenditure on infant mortality in sub-Saharan Africa: evidence from panel data analysis

**DOI:** 10.1186/s13561-021-00310-6

**Published:** 2021-03-27

**Authors:** Mwoya Byaro

**Affiliations:** IRDP, Research and Publication Unit, Mwanza, Tanzania

**Keywords:** Health care expenditures, Infant mortality, Bayesian framework, GMM

## Abstract

**Background:**

This commentary assesses critically the published article in the *Health Economics Review. 2020*; *10 (1), 1–9.* It explains the effects of health expenditure on infant mortality in sub-Saharan Africa using a panel data analysis (i.e. random effects) over the year 2000–2015 extracted from the World Bank Development Indicators. The paper is well written and deserve careful attention.

**Main text:**

The main reasons for inaccurate estimates observed in this paper are due to endogeneity issue with random effects panel estimators. It occurs when two or more variables simultaneously affect/cause each other. In this paper, the presence of endogeneity bias (i.e. education, health, health care expenditures and real GDP per capita variables) and its omitted variable bias leads to inaccurate estimates and conclusion. Random effects model require strict exogeneity of regressors. Moreover, frequentist/classic estimation (i.e. random effects) relies on sampling size and likelihood of the data in a specified model without considering other kinds of uncertainty.

**Conclusion:**

This comment argues future studies on health expenditures versus health outcomes (i.e. infant, under-five and neonates mortality) to use either dynamic panel (i.e. system Generalized Method of Moments, GMM) to control endogeneity issues among health (infant or neonates mortality), GDP per capita, education and health expenditures variables or adopting Bayesian framework to adjust uncertainty (i.e. confounding, measurement errors and endogeneity of variables) within a range of probability distribution.

## Background

There is a growing concern of the importance of population health and its contribution to the national economy, but the issue of infant mortality remains a major concern in most of the developing economies including Sub-Sahara Africa [1, 2]. One of the possible reasons for high infant mortality in sub-Saharan Africa could be low level of public health expenditure, low level of female education, poverty, poor sanitation, lack of safe drinking water and other basic utilities such as telecommunications and electricity [3]. This commentary aims to critique and correct the shortcomings observed in the panel data analysis about the effects of health care expenditures on infant mortality in 46 sub-Saharan African countries over the period 2000–2015. The paper is well written and deserves careful attention.

## Main text

The effects of health expenditure on infant mortality in sub-Saharan Africa has been published in *Health Economics Review, 10 (5)* by Kiross et al. [4], using macro data, relying on frequentist/classic methods. A study like this, among others preferred to use total health expenditure, public health expenditure and private health expenditure to explore its impact on life expectancy, infant mortality and under-five mortality, offering different conclusion (See, [1, 5–9]) with no consensus. The reasoning behind it is that, frequentist/classic estimation techniques depend entirely on sampling, and the likelihood of the data point given to the model without considering any kinds of uncertainty. Further, frequentist approach (i.e. random effects models) employed by Kiross et al. [4], among others (e.g., [1, 5]) leads in point estimates of parameter values, standard errors, CIs (confidence interval) and *P*-values arising from hypothesis tests (See, [10]). For instance, in authors article the *P*- value at 5% significance level shown by Kiross et al. [4] represent the probability that the data occurred in the specified model (i.e. random effect model) with the assumption that the null hypothesis is true and not false. Moreover, the overall model estimates relied on the coefficient of variables and fixing the values of parameters (i.e. ß as explanatory variables) using maximum likelihood leading to the final results and policy implication under uncertainty. Likewise, Kiross et al. [4] estimation ignored the true range of uncertainties (both model and parameter) and unobserved variables such as individual true disease, nutrition status and other confounding (i.e. number of physicians, corruption and misuse of public health funds) were not taken into account and are very common in sub-Saharan Africa. Kiross et al. [4] findings showed that, an increase in total health expenditure (external, public and private) was significant in reducing infant mortality in sub-Saharan Africa. Their findings was not clear whether the progress in decline of infant and neonatal mortality were primarily attributed to the increase/decrease of health care expenditures or other confounding factors.

Conversely, Kiross et al. [4] used health care expenditures (public or private), real GDP per capita, primary school enrollment rate as a proxy for education, population and other variables in the random effects regression as shown in. Nevertheless, the use of random effect models cannot overcome the problem of endogeneity issues (i.e. omitted variable bias, reverse causality) arising among health (i.e. infant mortality), education, GDP per capita and health care expenditures variables (See, [11, 12]). The endogeneity occurs when two or more variables simultaneously affect/cause each other. In other words; education, GDP per capita, health (i.e. infant or neonates mortality) and health expenditures variables in regression models may be correlated with the error term. This endogeneity bias can cause inconsistent estimates leading to misleading conclusion.

The endogeneity bias observed in Kiross et al. [4] paper can be eliminated by using the instrumental variables or two stages least square and system generalized methods of moments [13–16]. The Generalized Methods of Moments (GMM) is a known methodology to avoid the endogeneity bias by using instruments which are correlated with dependent variable and uncorrelated with the error term [14].

It is also known that countries with similar level of development like sub-Saharan Africa, public health spending differs significantly in health outcomes measures. For example, between 2010 and 2014, the average public health expenditure as percentage of GDP in Tanzania and Zambia was 2.5% and 2.5% respectively [17]. Similarly, infant mortality was higher in Zambia (48.8 per 1000 live births) than Tanzania (39.1 per 1000 live births). The key argument here is that, with the same level of public resources (i.e. 2.5% of GDP); one country can generates better health outcomes than another country. This also raises the question whether greater public health spending in sub-Saharan Africa suggested by Kiross et al. [4] can buy better health outcomes (i.e. reduce infant or neonatal mortality) or not. Wagstaff [18] argued that, if extra funds are likely to be applied extensively to health care, more staff at hospitals and adequate stocking of medications (i.e. panadol, amoxicillin etc) without complementary services (e.g. lack of roads networks to hospitals and clinics), the impact of extra public health expenditures on health outcomes (infants and neonates) as suggested by Kiross et al. [4] may be little or none. This implies that, increasing public health expenditures need to be complementary with spending in other sectors (water works, network of roads and education) to reduce both infant and neonatal mortality in sub-Sahara Africa. Such increases also need to be accompanied by policies, institutions, instruments (e.g., Public Expenditure Review and Management) and combating corruption (See, [18]). Based on the outlined facts above, it is clear that Kiross et al. [4] conclusion that, increasing government’s health care financing over the next years will be crucial in reducing mortality and improving health outcomes in sub-Sahara Africa still falls under uncertainty. This uncertainty may also occur due to the failure of random effects models to control endogeneity and other omitted variables bias.

## Conclusion

To address the aforementioned weakness encountered in Kiross et al. [4], the use of panel dynamic system Generalized method of moments (GMM) would be preferred to overcome endogeneity and its omitted variable bias present among health (infant or neonates mortality), real GDP per capita, education and health expenditures variables. Similarly, the use of Bayesian framework would be important for capturing the uncertainty of health expenditures (public and private) on infant mortality in Sub-Sahara Africa (See, [7]). The framework takes full account of uncertainties related to models, control confounding or unmeasured variables, and it uses decision making which is informed by both prior (i.e. hypothesis before observing the data) information and the new evidence obtained [10].

As a take home message for the readers and reviewers is that, random effects models require strict exogeneity of regressors and in the presence of endogeneity of variables, it leads to inaccurate estimates and misleading conclusion. Further, the Bayesian framework allows the authors to make use of prior knowledge or beliefs about the specific question being studied, as well as the new evidence collected specifically for the study [10]. It also enables the policy makers to use their own judgments about a sufficient level of evidence to make a policy decision [10]. The framework involves the probability that the true effect (i.e. the effects of health expenditure on infant mortality) falls into a particular range of values.

Future studies examining the effects of health expenditures (i.e. public or private) on health outcomes (i.e. infant and neonatal mortality) should either use dynamic system Generalized Methods of Moments (GMM) to control endogeneity and its omitted variables bias or adopting a Bayesian framework that provides a clear picture of parameter uncertainty adjusting for confounding, endogeneity and measurement error within a range of probability distribution (credible intervals).

## Data Availability

No dataset generated or analysed. The manuscript does contain any data. Not applicable for the commentary.

## Abbreviations

GMM: Generalized method of moments
GDP: Gross domestic product
